# Knowledge, Attitudes, Practices, and Perceptions of Occupational Health and Safety Among Healthcare Workers at a Tertiary Care Teaching Hospital in Jharkhand, India: A Cross-Sectional Study

**DOI:** 10.7759/cureus.116767

**Published:** 2026-09-23

**Authors:** Roma Kumari, Bhoopendra Singh, Anit Kujur

**Affiliations:** 1 Dentistry, Rajendra Institute of Medical Sciences, Ranchi, IND; 2 Forensic Medicine and Toxicology, Rajendra Institute of Medical Sciences, Ranchi, IND; 3 Hospital Administration, Rajendra Institute of Medical Sciences, Ranchi, IND; 4 Community Medicine, Rajendra Institute of Medical Sciences, Ranchi, IND

**Keywords:** healthcare workers, hepatitis b vaccination, knowledge-attitude-practice, occupational health, workplace safety

## Abstract

Background

Healthcare workers encounter biological, chemical, physical, ergonomic, and psychosocial hazards, yet evidence from heterogeneous hospital workforces in eastern India remains limited. This study evaluated occupational health and safety knowledge, attitudes, practices, and perceptions; quantified item-matched knowledge-practice gaps; and assessed self-reported hepatitis B vaccination uptake among healthcare workers at a tertiary care teaching hospital in Jharkhand.

Materials and methods

A hospital-based cross-sectional study was conducted from April 1 to June 30, 2026. Using stratified random sampling with equal allocation, 400 participants were enrolled: 100 nurses, 100 technician/paramedical staff, 100 housekeeping staff, and 100 ward attendants. An investigator-developed bilingual 32-item questionnaire, pilot-tested among 40 healthcare workers, measured knowledge (eight Yes/No items), attitude (five items), practice (five items, including a binary hepatitis B vaccination item), and perception (four items). Domain scores were converted to percentages and categorised using prespecified cutoffs. Categorical outcomes were compared using chi-square or exact tests, and four directly matched knowledge-practice pairs were evaluated using exact McNemar tests.

Results

The mean knowledge, attitude, practice, and perception percentages were 64.6%, 59.9%, 55.9%, and 53.9%, respectively. Poor practice was observed in 209 participants (52.2%; 95% CI, 47.4%-57.1%), unfavourable perception in 235 (58.8%; 95% CI, 53.9%-63.5%), and an overall poor/inadequate occupational health and safety profile in 227 (56.8%; 95% CI, 51.9%-61.5%). Knowledge-practice gaps were largest for occupational injury reporting (161/400, 40.2%), followed by personal protective equipment use (151/400, 37.8%); all four paired comparisons were significant (p<0.001). Recent safety training was associated with knowledge (p=0.002) and with attitude, practice, perception, and overall category (all p<0.001). Self-reported hepatitis B vaccination uptake was 41.5% (95% CI, 36.8%-46.4%).

Conclusion

The findings indicate an important gap between occupational safety knowledge and consistently reported safe practice, particularly for injury reporting, personal protective equipment use, hand hygiene, and biomedical waste segregation. Competency-based training, non-punitive exposure reporting, routine workplace audits, and accessible hepatitis B vaccination should prioritise support and outsourced workers while remaining available to all occupational groups.

## Introduction

Healthcare facilities protect patients but also expose workers to occupational risks. Healthcare workers may encounter blood and body fluids, infectious aerosols, hazardous chemicals, ionizing radiation, sharps, heavy manual handling, slips and falls, workplace violence, and psychosocial stress. The WHO and the International Labour Organization (ILO) therefore recommend integrated occupational health programmes that combine hazard prevention, worker training, surveillance, immunization, incident management, and organizational accountability [1].

The range and intensity of these exposures vary according to occupation and work setting. Clinical personnel may be more visible in occupational safety programmes, but housekeeping staff, ward attendants, and technical workers also handle contaminated surfaces, biomedical waste, equipment, and patient-care environments. Indian and international studies have documented incomplete awareness of occupational hazards, unsafe practices, and the influence of training, resource availability, and safety systems [2-5]. Aluko OO et al. reported high knowledge among Nigerian healthcare workers but substantially lower consistent adherence to standard procedures, illustrating that knowledge alone may not translate into safe behaviour [5].

Evidence combining knowledge, attitudes, self-reported practice, perceptions of the safety environment, and hepatitis B vaccination across clinical and support staff remains limited in Jharkhand. Survey-based research has greater scientific value when it identifies actionable behavioural gaps rather than only reporting aggregate awareness. Accordingly, this study aimed to (1) assess occupational health and safety knowledge, attitudes, practices, and perceptions among four categories of healthcare workers; (2) quantify gaps between correct knowledge and consistently reported practice for directly corresponding safety behaviours; (3) estimate self-reported hepatitis B vaccination uptake; and (4) develop institutionally relevant recommendations. No directional hypotheses were prespecified; the association analyses were exploratory.

## Materials and methods

Study design and setting

This hospital-based cross-sectional observational study was conducted at Rajendra Institute of Medical Sciences (RIMS), Ranchi, Jharkhand, India, from April 1 to June 30, 2026. RIMS is a tertiary care teaching hospital serving patients from Jharkhand and neighbouring regions.

This report was prepared in accordance with the Strengthening the Reporting of Observational Studies in Epidemiology (STROBE) recommendations for cross-sectional studies [6].

Study population and eligibility

The study population comprised nurses, technician/paramedical personnel, housekeeping staff, and ward attendants. Staff members were eligible if they had worked at the institution for at least six months, were available during the data-collection period, were willing to participate, and provided written informed consent. Staff employed for less than six months, those on long-term leave or otherwise unavailable during data collection, and those who declined participation were excluded.

Sample size and sampling

The minimum sample size was calculated using the single-population-proportion formula, n=Z²p(1-p)/d². At a 95% confidence level (Z=1.96), an anticipated proportion of 71% selected with reference to the study by Aluko OO et al. [5], and 5% absolute precision, the calculated sample size was approximately 317. After adding 10% for possible non-response, the target was approximately 349; this was increased to 400 to obtain equal representation from the four occupational strata.

Disproportionate stratified random sampling was used. The sampling frame was divided into four occupational strata, and 100 participants were selected from each stratum by simple random sampling. Equal allocation was chosen to support comparisons across occupational groups; therefore, overall percentages describe the study sample and should not be interpreted as workforce-weighted institutional prevalence unless the institutional workforce has the same occupational composition.

Questionnaire and study measures

Data were collected using a structured, closed-ended, bilingual (English/Hindi), investigator-developed questionnaire that was not adopted or adapted from any previously published instrument. The 32-item questionnaire comprised five sections: Section A, 10 socioeconomic and work-related variables; Section B, eight Yes/No knowledge items (K11-K18) on occupational safety; Section C, five attitude items; Section D, four frequency-based practice items and one hepatitis B vaccination item (P26); and Section E, four perception items (Appendices). The prespecified outcomes were knowledge, attitude, practice, perception, and overall occupational health and safety scores and categories; four item-matched knowledge-practice gaps; and self-reported hepatitis B vaccination uptake. Occupational group and occupational safety training during the preceding year were the principal explanatory variables. Age, gender, education, employment status, work area, experience, family income, and residence were recorded as participant characteristics and potential confounders. No adjusted causal model was prespecified. All occupational groups were assessed using the same questionnaire, response definitions, and scoring procedures.

Questionnaire validation and data collection

Subject experts reviewed the questionnaire for relevance, clarity, and content coverage, and it was pretested before the main survey to assess comprehensibility and feasibility. Individuals involved in pretesting were not included in the final analysis. Eligible staff were approached in their work areas after administrative approval. The study purpose was explained, written informed consent was obtained, and no participant names were recorded. Completed questionnaires were checked for completeness and internal consistency before data entry.

Measures to address bias

Selection bias was addressed through occupation-stratified simple random sampling from the available sampling frame and the use of uniform eligibility criteria. Measurement variation across groups was reduced by using the same pretested bilingual instrument, response options, scoring rules, and data-collection procedures. The questionnaire was anonymous to reduce social-desirability concerns, and completed forms were checked before data entry. Duplicate, missing, and out-of-range values were assessed during data cleaning. Interviewer blinding was not applicable, and no validation against observation, employment records, vaccination documents, or serology was performed.

Scoring and interpretation

Each knowledge item was scored Yes=1 and No=0, producing a knowledge score of 0-8. Attitude items were scored Agree=2, Not sure=1, and Disagree=0, producing a score of 0-10. Practice items P24, P25, P27, and P28 were scored Always=2, Sometimes=1, and Never=0. The hepatitis B vaccination item P26 was scored Yes=2 and No=0; the practice score therefore ranged from 0 to 10. Perception items were scored Agree=2, Not sure=1, and Disagree=0, producing a score of 0-8. The overall score ranged from 0 to 36.

Raw scores were converted to percentages. Scores of 80-100%, 60-79.9%, and <60% were categorised, respectively, as good, average, and poor knowledge; favourable, neutral, and unfavourable attitude; good, moderate, and poor practice; favourable, average, and unfavourable perception; and good/adequate, moderate/average, and poor/inadequate overall occupational health and safety status.

Knowledge-practice gaps and hepatitis B vaccination uptake

Four directly corresponding item pairs were evaluated: knowledge that handwashing prevents infection versus reported handwashing before and after work; knowledge that personal protective equipment (PPE) should be selected according to work type versus reported PPE use; knowledge that occupational injuries should be reported versus reported notification of a supervisor after injury; and knowledge of correct colour-coded waste segregation versus reported disposal in the correct bin. A knowledge-practice gap was defined as a correct knowledge response (Yes) accompanied by a practice response of Sometimes or Never. An Always response represented consistent practice. No Not applicable responses were present in the final analytical dataset.

Self-reported hepatitis B vaccination uptake was assessed using P26. A Yes response indicated reported uptake, and a No response indicated no reported uptake. The item did not measure the number of doses, schedule completion, documentary verification, post-vaccination antibody titres, or barriers to vaccination.

Data management and statistical analysis

Questionnaires were assigned respondent identification numbers and entered into Microsoft Excel (Microsoft Corporation, Redmond, Washington, United States). The final dataset was checked for duplicate identifiers, missing values, valid coding ranges, and agreement between responses and calculated domain scores. All 400 records were complete, and no imputation was required.

Statistical analysis was performed using Python 3.12.13, pandas 2.2.3, and SciPy 1.17.0. Categorical variables are reported as frequencies and percentages; domain and overall scores are reported as mean ± SD. Wilson 95% CIs were calculated for principal proportions. Pearson chi-square tests assessed unadjusted associations of occupational category and recent safety training with categorical outcomes, with continuity correction applied to the 2×2 perception table. Expected counts were examined. For the 2×3 association between training and overall occupational health and safety category, the chi-square assumption was not met; therefore, the Fisher-Freeman-Halton exact test was performed using Monte Carlo estimation with 1,000,000 samples and a fixed random seed of 20260821. Paired knowledge and practice responses were compared using two-sided exact McNemar tests, implemented as exact binomial tests of discordant pairs. All tests were two-sided, p<0.05 was considered statistically significant, and association analyses were interpreted as exploratory because no adjustment was made for multiple comparisons. No multivariable adjustment, interaction modelling, sampling weights, or sensitivity analyses were prespecified or performed. Because equal numbers were sampled from each occupational stratum, overall estimates describe the study sample and not a workforce-weighted institutional population.

Ethical considerations

The study received final approval from the Institutional Ethics Committee, RIMS, Ranchi, Jharkhand, India (IEC Registration No. ECR/769/INST/JH/2015/RR-21; Approval Letter No. 60, dated February 24, 2026; Synopsis Submission No. S/50/26). Participation was voluntary, written informed consent was obtained from all participants, and no names or direct personal identifiers were recorded. Confidentiality was maintained throughout data collection, analysis, and reporting, and the information was used only for research purposes.

## Results

All 400 completed records in the final dataset were included in the analysis. There were no missing values or duplicate respondent identifiers, and each occupational group contributed 100 participants. A separate screening log recording the numbers approached, unavailable, excluded before questionnaire completion, or declining participation was not available; therefore, the response rate and stage-specific non-participation could not be calculated, and a detailed recruitment flow diagram could not be prepared.

Participant characteristics

The largest age group was 26-35 years (166/400, 41.5%), followed by 36-45 years (124/400, 31.0%). Females accounted for 205 participants (51.2%). Half of the sample was employed through outsourcing arrangements (201/400, 50.2%), and 238 participants (59.5%) reported receiving occupational safety training during the preceding year (Table 1).

**Table 1:** Participant characteristics (N = 400). INR: Indian rupee.

Variables	Category	n	%
Age group, years	18-25	56	14
	26-35	166	41.5
	36-45	124	31
	>45	54	13.5
Gender	Female	205	51.2
	Male	195	48.8
Education	No formal education	12	3
	Primary	42	10.5
	High school	107	26.8
	Intermediate	42	10.5
	Graduate	58	14.5
	Postgraduate	139	34.8
Employment status	Permanent	122	30.5
	Contractual	76	19
	Outsourced	201	50.2
	Trainee/intern	1	0.2
Work area	Ward	118	29.5
	ICU/operation theatre	69	17.2
	Outpatient department	112	28
	Laboratory	22	5.5
	Other	79	19.8
Work experience, years	<1	31	7.8
	1-5	152	38
	6-10	120	30
	>10	97	24.2
Monthly family income	INR <20,000	154	38.5
	INR 20,001-30,000	68	17
	INR 30,001-40,000	73	18.2
	INR 40,001-50,000	24	6
	>INR 50,000	65	16.2
	No response	16	4
Residence	Rural	160	40
	Urban	156	39
	Semi-urban	84	21
Safety training in the preceding year	Yes	238	59.5
	No	162	40.5

Knowledge, attitude, practice, perception, and overall scores

The mean knowledge score was 5.17±1.58 out of 8 (64.6%); 195 participants (48.8%) had average knowledge, and 81 (20.2%) had good knowledge. The mean attitude score was 5.99±1.23 out of 10 (59.9%), and only 48 participants (12.0%) had a favourable attitude. The mean practice score was 5.59±1.44 out of 10 (55.9%); 209 participants had poor practice (52.2%; 95% CI, 47.4%-57.1%). The mean perception score was 4.32±1.06 out of 8 (53.9%), and no participant reached the favourable-perception category; 235 had an unfavourable perception (58.8%; 95% CI, 53.9%-63.5%). The mean overall score was 21.06±4.22 out of 36 (58.5%), with 227 participants categorised as poor/inadequate (56.8%; 95% CI, 51.9%-61.5%) (Table 2).

**Table 2:** Domain and overall score distributions (N = 400). Percentages may not total exactly 100.0% because of rounding.

Domain	Range	Mean ± SD	Mean (%)	High category, n (%)	Middle category, n (%)	Low category, n (%)
Knowledge	0-8	5.17 ± 1.58	64.6	Good: 81 (20.2)	Average: 195 (48.8)	Poor: 124 (31.0)
Attitude	0-10	5.99 ± 1.23	59.9	Favourable: 48 (12.0)	Neutral: 200 (50.0)	Unfavourable: 152 (38.0)
Practice	0-10	5.59 ± 1.44	55.9	Good: 50 (12.5)	Moderate: 141 (35.2)	Poor: 209 (52.2)
Perception	0-8	4.32 ± 1.06	53.9	Favourable: 0 (0.0)	Average: 165 (41.2)	Unfavourable: 235 (58.8)
Overall	0-36	21.06 ± 4.22	58.5	Good/adequate: 7 (1.8)	Moderate/average: 166 (41.5)	Poor/inadequate: 227 (56.8)

Knowledge-practice gaps

For every matched item, the proportion reporting consistent practice was lower than the proportion with correct knowledge. The largest gap concerned occupational injury reporting: 161 participants (40.2% of the full sample; 95% CI, 35.6%-45.1%), representing 61.9% of those with correct knowledge, knew that injuries should be reported but reported notifying a supervisor only sometimes or never. Gaps were also present for PPE use (151/400, 37.8%; 95% CI, 33.1%-42.6%), hand hygiene (136/400, 34.0%; 95% CI, 29.5%-38.8%), and biomedical waste segregation (136/400, 34.0%; 95% CI, 29.5%-38.8%). All four exact McNemar tests were significant (p<0.001) (Table 3).

**Table 3:** Item-specific knowledge-practice gaps (N = 400). A gap was defined as correct knowledge (“Yes”) with practice reported as “Sometimes” or “Never.” P-values are from two-sided exact McNemar tests.

Matched item	Knowledge: Yes, n (%)	Practice: Always, n (%)	Gap, n (% of N)	Gap among knowledgeable, %	Exact p-value
Hand hygiene	259 (64.8)	164 (41.0)	136 (34.0)	52.5	<0.001
Personal protective equipment	258 (64.5)	163 (40.8)	151 (37.8)	58.5	<0.001
Occupational injury reporting	260 (65.0)	153 (38.2)	161 (40.2)	61.9	<0.001
Biomedical waste segregation	255 (63.7)	180 (45.0)	136 (34.0)	53.3	<0.001

Domain levels by occupational group

Domain categories differed across occupations (all p<0.001). Nurses had the most favourable profile: 48.0% had good knowledge, 48.0% had a favourable attitude, 50.0% had good practice, and all had average perception. In contrast, 70.0% of housekeeping staff had poor knowledge, and all housekeeping staff were categorised as having an unfavourable attitude, poor practice, and unfavourable perception. Ward attendants also had substantial deficits, including poor practice in 69.0% and unfavourable perception in 100% (Table 4).

**Table 4:** Domain-level categories by occupational group. Pearson chi-square tests comparing occupational groups: knowledge: χ² = 155.39, df = 6; attitude: χ² = 377.20, df = 6; practice: χ² = 313.44, df = 6; perception: χ² = 306.13, df = 3; all p < 0.001.

Domain	Occupational group	Good/favourable, n (%)	Average/neutral/moderate, n (%)	Poor/unfavourable, n (%)
Knowledge	Nurse	48 (48.0)	47 (47.0)	5 (5.0)
	Technician/paramedical	22 (22.0)	63 (63.0)	15 (15.0)
	Housekeeping	3 (3.0)	27 (27.0)	70 (70.0)
	Ward attendant	8 (8.0)	58 (58.0)	34 (34.0)
Attitude	Nurse	48 (48.0)	52 (52.0)	0 (0.0)
	Technician/paramedical	0 (0.0)	86 (86.0)	14 (14.0)
	Housekeeping	0 (0.0)	0 (0.0)	100 (100.0)
	Ward attendant	0 (0.0)	62 (62.0)	38 (38.0)
Practice	Nurse	50 (50.0)	50 (50.0)	0 (0.0)
	Technician/paramedical	0 (0.0)	60 (60.0)	40 (40.0)
	Housekeeping	0 (0.0)	0 (0.0)	100 (100.0)
	Ward attendant	0 (0.0)	31 (31.0)	69 (69.0)
Perception	Nurse	0 (0.0)	100 (100.0)	0 (0.0)
	Technician/paramedical	0 (0.0)	65 (65.0)	35 (35.0)
	Housekeeping	0 (0.0)	0 (0.0)	100 (100.0)
	Ward attendant	0 (0.0)	0 (0.0)	100 (100.0)

Association with occupational safety training

Participants who had received occupational safety training during the preceding year had more favourable category distributions across all measured domains. Good practice was observed in 37 of 238 trained participants (15.5%) and 13 of 162 untrained participants (8.0%), while poor practice was observed in 104 (43.7%) and 105 (64.8%), respectively. Training was associated with knowledge (p=0.002) and with attitude, practice, perception, and overall category (all p<0.001) (Table 5). These cross-sectional associations do not establish that training caused the observed differences.

**Table 5:** Association of occupational safety training with domain and overall categories. Pearson chi-square tests (with continuity correction for the 2×2 perception table) were used for knowledge, attitude, practice, and perception; associations were statistically significant (p = 0.002 for knowledge, p < 0.001 for attitude, practice, and perception). The Fisher-Freeman-Halton exact test was used for the overall category (p < 0.001).

Outcome	Category	Training: Yes, n (%)	Training: No, n (%)	Test statistic	p-value
Knowledge	Good	61 (25.6)	20 (12.3)	12.38	0.002
	Average	114 (47.9)	81 (50.0)		
	Poor	63 (26.5)	61 (37.7)		
Attitude	Favourable	36 (15.1)	12 (7.4)	13.85	<0.001
	Neutral	128 (53.8)	72 (44.4)		
	Unfavourable	74 (31.1)	78 (48.1)		
Practice	Good	37 (15.5)	13 (8.0)	17.64	<0.001
	Moderate	97 (40.8)	44 (27.2)		
	Poor	104 (43.7)	105 (64.8)		
Perception	Average	122 (51.3)	43 (26.5)	23.29	<0.001
	Unfavourable	116 (48.7)	119 (73.5)		
Overall	Good/adequate	6 (2.5)	1 (0.6)	Exact	<0.001
	Moderate/average	126 (52.9)	40 (24.7)		
	Poor/inadequate	106 (44.5)	121 (74.7)		

Hepatitis B vaccination uptake

Hepatitis B vaccination uptake was reported by 166 participants (41.5%; 95% CI, 36.8%-46.4%); 234 (58.5%) responded “No.” Reported uptake was highest among nurses (64.0%), followed by technician/paramedical staff (46.0%), ward attendants (30.0%), and housekeeping staff (26.0%) (Table 6). These values represent self-reported uptake and not verified completion of a vaccination schedule or serological protection.

**Table 6:** Self-reported hepatitis B vaccination uptake by occupational group. The questionnaire item asked, “Have you taken the hepatitis B vaccine?” It did not assess dose completion or anti-HBs titre. Anti-HBs: Antibody to hepatitis B surface antigen.

Occupational group	Yes, n (%)	No, n (%)	Total
Nurse	64 (64.0)	36 (36.0)	100
Technician/paramedical staff	46 (46.0)	54 (54.0)	100
Housekeeping staff	26 (26.0)	74 (74.0)	100
Ward attendant	30 (30.0)	70 (70.0)	100
Total	166 (41.5)	234 (58.5)	400

## Discussion

Principal findings

This cross-sectional study identified substantial occupational health and safety deficiencies across a heterogeneous hospital workforce. Knowledge was in the average range (mean percentage, 64.6%), while the mean attitude, practice, and perception percentages were 59.9%, 55.9%, and 53.9%, respectively. More than half of the participants had poor practice, unfavourable perception, and an overall poor/inadequate profile. The principal operational concern is therefore not limited knowledge alone, but the inconsistent translation of knowledge into routine safety behaviour.

The occupational pattern was marked. Nurses had the most favourable profile, whereas housekeeping staff and ward attendants showed the largest deficits. A tertiary care study from Jharkhand similarly reported professional-group variation in sterilization-related knowledge, attitudes, and practices [7]. In contrast, Joshi P et al. found good overall infection-control knowledge, attitude, and practice in 69.2% of housekeeping and sanitation workers in an eastern Indian tertiary hospital [8]. Such differences suggest that findings cannot be generalised across institutions without considering induction, supervision, work allocation, employment arrangements, and access to safety resources.

Knowledge, attitude, practice, and perception

Only 20.2% of participants demonstrated good occupational health and safety knowledge, while 31.0% had poor knowledge. Clinical and technical staff performed better than support workers. Formal professional education and repeated exposure to infection-prevention protocols may partly explain this pattern, but support workers often handle contaminated surfaces, waste, and inappropriately discarded sharps. Their lower scores therefore represent a direct occupational vulnerability rather than a peripheral training concern.

Only 12.0% of participants had a favourable attitude, whereas 50.0% were neutral and 38.0% were unfavourable. Neutrality may reflect uncertainty about the importance or feasibility of recommended precautions, although the questionnaire did not assess the reasons for this. Odonkor ST and Sallar AM reported high occupational safety knowledge but weaker implementation in Ghana [9]. Liu Y et al. found that nurses’ preventive practices were associated with both knowledge and attitude and highlighted the roles of training and work experience [10]. These findings support a systems interpretation in which education is necessary, but behaviour is also shaped by supervision, supply availability, workload, safety climate, and consequences for non-adherence.

Practice was the weakest actionable domain: 52.2% of participants had poor practice, and only 12.5% had good practice. The occupational gradient suggests that a single uniform lecture is unlikely to correct the problem. Support staff may require language-appropriate demonstrations, return demonstrations, repeated observation, and supervisor feedback. Clinical staff may benefit from audit-based reinforcement of exposure reporting, PPE selection, and hand hygiene. Gandhi P et al. reported generally good PPE practices among intensive care nurses but still identified remediable gaps and recommended periodic reinforcement and teamwork [11].

Knowledge-practice gaps

The matched analysis showed a significant gap between knowing the recommended action and reporting that it was always performed. Occupational injury reporting had the largest gap: 40.2% of all participants and 61.9% of those with correct knowledge reported notifying a supervisor only sometimes or never. Failure to report exposure incidents can delay risk assessment, post-exposure prophylaxis where indicated, documentation, and corrective action. In a survey of more than 7,000 healthcare workers in China, 28.53% of needlestick and sharps injuries were unreported [12]. Sain S et al. likewise emphasised the need for strong knowledge and practices concerning needlestick injuries and the prevention of HIV and hepatitis B transmission [13].

Important gaps were also observed for PPE use (37.8% of all participants), hand hygiene (34.0%), and biomedical waste segregation (34.0%). In every pair, more than half of the participants with correct knowledge did not report consistent practice. Diwan T et al. documented variable biomedical waste knowledge, attitudes, and practices among healthcare workers in Chhattisgarh [14]. These findings indicate that improvement programmes should measure observable performance and work-system barriers rather than relying only on post-training knowledge scores.

Occupational safety training

Recent safety training was associated with every measured domain. Good practice was reported by 15.5% of trained participants and 8.0% of untrained participants; poor practice occurred in 43.7% and 64.8%, respectively. This supports the relevance of training but does not establish causation because exposure to training was not randomised, and departments with stronger safety systems may be more likely both to train workers and to reinforce safe behaviour.

Training should be implemented as a continuing competency programme rather than an attendance event. Demonstrations and return demonstrations should cover PPE selection, hand hygiene, waste segregation, safe lifting, and exposure reporting. The WHO and ILO recommend comprehensive programmes integrating prevention, training, surveillance, immunization, incident management, and organizational responsibility [1]. Outsourced and support workers should be included on the same basis as permanent clinical staff.

Hepatitis B vaccination uptake

Self-reported hepatitis B vaccination uptake was 41.5%, indicating that most participants responded “No.” Uptake ranged from 64.0% among nurses to 26.0% among housekeeping staff. This was lower than the 70.32% complete vaccination coverage reported among healthcare workers in South Delhi [15]. The comparison should be interpreted cautiously because the present P26 item measured only a binary self-report and did not establish the number of doses or completion of a recommended schedule.

Vaccination history and serological protection are not equivalent. Konda V and Vijayaraghavan S measured anti-HBs titres among healthcare workers and highlighted the value of post-vaccination assessment and identification of non-responders [16]. The present finding should therefore be described as self-reported vaccination uptake, not confirmed complete vaccination or immunity. Because reasons for non-uptake were not assessed, cost, access, fear, beliefs, vaccine availability, and scheduling difficulty cannot be presented as observed barriers.

Implications for practice

The findings support a risk-stratified occupational health programme. Housekeeping staff, ward attendants, outsourced workers, and personnel without recent safety training should be prioritised without excluding clinical staff. Induction should occur before workplace deployment and be followed by periodic competency assessment. Department-level audits should monitor hand hygiene, PPE selection and use, colour-coded waste segregation, and occupational exposure reporting. Supervisors should provide immediate feedback, while recurrent non-adherence should trigger investigation of workflow, supplies, staffing, and environmental constraints rather than being attributed solely to individual behaviour.

A confidential occupational health registry should document training, exposure incidents, post-exposure management, hepatitis B vaccine doses, and anti-HBs testing where indicated. Vaccination should be accessible during duty hours and should cover outsourced and support personnel. Because injury reporting showed the largest knowledge-practice gap, the hospital should establish a simple, non-punitive reporting pathway linked to rapid post-exposure assessment. Repeat audits using the same definitions could evaluate whether improvements are sustained.

Strengths and limitations

The study included 400 participants with equal representation from four occupational groups and assessed knowledge, attitude, practice, perception, safety training, and hepatitis B vaccination in the same workforce. Its item-matched analysis quantified specific knowledge-practice gaps rather than relying solely on aggregate scores.

Several limitations should be considered. The cross-sectional design does not establish temporal or causal relationships. All measures, including practices, training, and vaccination, were self-reported. Social-desirability bias would most plausibly inflate favourable practice, training, and vaccination estimates, whereas recall error could bias estimates in either direction. Because a separate screening log was not available, the response rate and the extent and direction of non-participation bias could not be quantified. Equal allocation supports group comparison but may overestimate or underestimate hospital-wide proportions depending on the actual occupational composition of the workforce. Occupation is closely related to education, employment arrangement, income, and gender; unadjusted associations may therefore reflect confounding and cannot be interpreted as independent effects. The binary training item did not measure duration, content, quality, frequency, or competency. P26 did not document vaccine-dose completion, records, seroprotection, or reasons for non-uptake. The questionnaire's psychometric properties were not established beyond expert review and pretesting.

Generalisability

The findings are directly applicable to the 400-person study sample and may inform occupational health planning in similar tertiary teaching hospitals with mixed clinical and support workforces. They should not be interpreted as workforce-weighted prevalence estimates for RIMS, Ranchi, or generalised to other institutions without considering differences in workforce composition, contracting arrangements, training systems, safety resources, and local work practices. The single-centre setting further limits external validity.

## Conclusions

Among 400 healthcare workers at a tertiary care teaching hospital in Jharkhand, knowledge was generally average, but practice, perception, and overall occupational health and safety profiles were frequently inadequate. The largest knowledge-practice gap involved occupational injury reporting, followed by PPE use, hand hygiene, and biomedical waste segregation. Recent training was associated with more favourable outcomes, although causal inference is not possible. Self-reported hepatitis B vaccination uptake was low, particularly among support workers. The hospital should implement competency-based induction and refresher training, observable-practice audits, non-punitive exposure reporting, and accessible hepatitis B vaccination and follow-up for all staff, with particular attention to housekeeping staff, ward attendants, and outsourced personnel.

## Appendices

**Table 7:** Questionnaire used in the study. OT: Operation theatre; PPE: Personal protective equipment.

Item no.	Question/item	Response options
Section A: Socioeconomic and Work Details | अनुभाग A: सामाजिक-आर्थिक एवं कार्य संबंधी जानकारी		
1	Age आयु	18-25 / 26-35 / 36-45 / >45 18-25 / 26-35 / 36-45 / 45 से अधिक
2	Gender लिंग	Male / Female / Other पुरुष / महिला / अन्य
3	Education शिक्षा	No formal / Primary / High school / Intermediate / Graduate / Postgraduate औपचारिक नहीं / प्राथमिक / हाई स्कूल / इंटरमीडिएट / स्नातक / परास्नातक
4	Job category कार्य का प्रकार	Doctor / Nurse / Technician / Housekeeping / Ward attendant / Other डॉक्टर / नर्स / टेक्नीशियन / सफाई कर्मचारी / वार्ड अटेंडेंट / अन्य
5	Employment नौकरी	Permanent / Contractual / Outsourced / Trainee/Intern स्थायी / संविदा / आउटसोर्स्ड / प्रशिक्षु/इंटर्न
6	Work area कार्य क्षेत्र	Ward / ICU/OT / OPD / Lab / Cleaning area / Other वार्ड / ICU/OT / OPD / लैब / सफाई क्षेत्र / अन्य
7	Work experience कार्य अनुभव	<1 yr / 1-5 yr / 6-10 yr / >10 yr 1 वर्ष से कम / 1-5 वर्ष / 6-10 वर्ष / 10 वर्ष से अधिक
8	Monthly family income परिवार की मासिक आय	<20,000 / 20,001-30,000 / 30,001-40,000 / 40,001-50,000 / >50,000 / No reply <20,000 / 20,001-30,000 / 30,001-40,000 / 40,001-50,000 / 50,000 से अधिक / नहीं बताना
9	Residence निवास	Rural / Urban / Semi-urban ग्रामीण / शहरी / अर्धशहरी
10	Have you received infection control and safety training in the last 1 year? पिछले 1 वर्ष में इन्फेक्शन कंट्रोल और सेफ्टी की ट्रेनिंग मिली है?	Yes / No हाँ / नहीं
Section B: Knowledge Questions | अनुभाग B: ज्ञान आधारित प्रश्न		
11	Have you heard about workplace safety? क्या आपने कार्यस्थल सुरक्षा के बारे में सुना है?	Yes / No हाँ / नहीं
12	Hand washing prevents infection. हाथ धोने से संक्रमण से बचाव होता है।	Yes / No हाँ / नहीं
13	Disease can spread through blood/body fluids. खून/शरीर के तरल पदार्थों से बीमारी फैल सकती है।	Yes / No हाँ / नहीं
14	PPE should be used according to work type. काम के अनुसार PPE का उपयोग करना चाहिए।	Yes / No हाँ / नहीं
15	Needlestick/sharps injury can spread infection. सुई/नुकीली चीज की चोट से संक्रमण फैल सकता है।	Yes / No हाँ / नहीं
16	Hospital waste should be disposed of in the correct colour-coded bin. अस्पताल का कचरा सही रंग के डिब्बे में डालना चाहिए।	Yes / No हाँ / नहीं
17	Injury at work should be reported. काम के दौरान चोट की सूचना देना जरूरी है।	Yes / No हाँ / नहीं
18	Ergonomics: Lift heavy items by bending the knees and keeping the back straight. एर्गोनॉमिक्स: भारी सामान घुटने मोड़कर और पीठ सीधी रखकर उठाना सुरक्षित है।	Yes / No हाँ / नहीं
Section C: Attitude Questions | अनुभाग C: दृष्टिकोण आधारित प्रश्न		
19	Safety at work is important for every staff member. कार्यस्थल सुरक्षा हर कर्मचारी के लिए महत्वपूर्ण है।	Agree / Not sure / Disagree सहमत / निश्चित नहीं / असहमत
20	Wearing PPE when needed is necessary. जरूरत पड़ने पर PPE पहनना आवश्यक है।	Agree / Not sure / Disagree सहमत / निश्चित नहीं / असहमत
21	Safety training should be given regularly. सुरक्षा प्रशिक्षण नियमित रूप से दिया जाना चाहिए।	Agree / Not sure / Disagree सहमत / निश्चित नहीं / असहमत
22	Work injuries should be reported immediately. काम के दौरान लगी चोट की सूचना तुरंत देनी चाहिए।	Agree / Not sure / Disagree सहमत / निश्चित नहीं / असहमत
23	Waste segregation is the responsibility of all staff. कचरे को अलग करना सभी कर्मचारियों की जिम्मेदारी है।	Agree / Not sure / Disagree सहमत / निश्चित नहीं / असहमत
Section D: Practice Questions | अनुभाग D: व्यवहार/अभ्यास आधारित प्रश्न		
24	Do you wash your hands before and after work? क्या आप काम से पहले और बाद में हाथ धोते हैं?	Always / Sometimes / Never हमेशा / कभी-कभी / कभी नहीं
25	Do you use the required PPE, such as gloves, masks, aprons, or boots? क्या आप जरूरी PPE जैसे दस्ताने/मास्क/एप्रन/जूते पहनते हैं?	Always / Sometimes / Never हमेशा / कभी-कभी / कभी नहीं
26	Have you received the hepatitis B vaccine? क्या आपने हेपेटाइटिस-B का टीका लगवाया है?	Yes / No हाँ / नहीं
27	If injured at work, do you inform your supervisor? काम के दौरान चोट लगने पर क्या आप वरिष्ठ को बताते हैं?	Always / Sometimes / Never / Not Applicable हमेशा / कभी-कभी / कभी नहीं / लागू नहीं
28	Do you put hospital waste in the correct bin? क्या आप अस्पताल का कचरा सही डिब्बे में डालते हैं?	Always / Sometimes / Never हमेशा / कभी-कभी / कभी नहीं
Section E: Perception Questions | अनुभाग E: धारणा आधारित प्रश्न		
29	PPE and safety materials are available in my work area. मेरे कार्यक्षेत्र में PPE और सुरक्षा सामग्री उपलब्ध रहती है।	Agree / Not sure / Disagree सहमत / निश्चित नहीं / असहमत
30	Safety instructions/signboards are easy to understand. सुरक्षा निर्देश/बोर्ड समझने में आसान हैं।	Agree / Not sure / Disagree सहमत / निश्चित नहीं / असहमत
31	I feel safe while doing my daily hospital work. अस्पताल में रोज का काम करते समय मुझे सुरक्षित महसूस होता है।	Agree / Not sure / Disagree सहमत / निश्चित नहीं / असहमत
32	My supervisor supports me in following safety rules and reporting injuries. वरिष्ठ सुरक्षा नियमों का पालन और चोट की सूचना देने में सहयोग करते हैं।	Agree / Not sure / Disagree सहमत / निश्चित नहीं / असहमत
